# AgroDrug Conjugates for Sustainable Crop Protection: Molecular Architectures, Mechanisms, and Critical Perspectives

**DOI:** 10.1007/s41061-025-00531-x

**Published:** 2025-11-14

**Authors:** Giulia Cazzaniga, Roberto Orru, David M. Barber, Silvia Gazzola

**Affiliations:** 1 Department of Science and High Technology, University of Insubria, Via Valleggio 9, 22100 Como, Italy; 2 Research and Development, Weed Control, Bayer AG, Crop Science Division, Industriepark Höchst, 65926 Frankfurt Am Main, Germany

**Keywords:** Agrochemicals, Agrodrugs, AgroDrug conjugates, Crop protection, Sustainable agriculture

## Abstract

The agriculture sector faces significant challenges from weeds and pests, exacerbated by climate change. Traditional control methods have led to the emergence of difficult to manage resistant populations, threatening global food security. AgroDrug conjugates (AgDCs) offer a promising approach to enhance agrodrug bioavailability and systemic distribution within plant tissues. This can be accomplished by attaching agrodrugs to molecular carriers such as sugars or amino acids. AgDCs aim to improve targeting and efficiency, while reducing the environmental impact. This review seeks to deliver a thorough and critical analysis of the chemical architectures and underlying mechanisms of action of AgDCs as documented in current scientific literature. Moreover, we highlight advances and knowledge gaps in AgDC design, including metabolic stability, ecological safety, and field-scale performance. Addressing these challenges will be essential to unlock the full potential of AgDCs as next-generation tools for sustainable and resilient crop protection.

## Introduction

The agriculture sector represents a key contributor of the global economy, serving as a primary source of food and income for millions worldwide. However, food production in farming systems is continuously threatened by unwanted weeds and pests, which can cause yield losses of more than 45% in crop production [1]. Their persistence and high adaptability to environmental changes pose relevant problems for food security and financial drawbacks to farmers [2], which are additionally affected by climate change that is currently causing increased average temperatures, higher risks of extreme natural events, and land degradation processes in the whole of Europe and across the world. In the last century, the use of manufactured weed and pest control strategies effectively allowed humans to enhance the efficiency of arable fields, but the increase of weed and pest populations that are resistant to these compounds is currently alarming the agriculture sector. In addition, according to the Food and Agriculture Organization (FAO), the worldwide population is expected to reach over 9 billion by 2050, and consequently, it is estimated that the world demand for food will increase by up to 70% [3]. The current crop production industry is not sufficient to feed the rising population, also considering that agriculture is expected to provide subsistence for about half the population in the developing countries by 2050. Considering that there is still a heavy reliance on the use of fertilizers and agrodrugs in the agricultural industry, which impairs the ecological status of the environment, the implementation of sustainability aspects in agriculture is thus recognized as a primary challenge for the development policies planned by the European Union (EU). Indeed, in June 2022, the official request to reduce the use of chemical crop protection products by 50% before 2030 had been introduced in the “Sustainable Use Regulation” that formed part of the “Green Deal” proposal, designed to protect the environment and to reverse the degradation of the ecosystem [4]. However, the challenges in complying with the new EU regulations in the agriculture sector led to significant protests by farmers, resulting in the subsequent withdrawal of the proposal by the EU Commission. While this may be seen as a setback in achieving sustainable agriculture in the EU, it also highlights the agricultural community’s demand for efficient crop protection products that can ensure a positive, sustainable environmental and economic impact [5]. New technologies are currently being developed to discover novel agrodrugs with different modes of action. This research field is essential, but it takes a long time for a new chemical entity to be approved and commercialized [6]. For this reason, developing new and efficient systems to repurpose existing herbicides that are currently not very effective is an attractive and growing area of research in this field [7]. In this context, one of the main issues faced by some traditional agrodrugs, particularly those that have not been successfully developed, is their inefficient ability to be absorbed and distributed throughout different plant tissues after application. Inefficiencies in bioavailability necessitate higher dosages, which not only raise concerns about environmental impact and crop damage but may also diminish the likelihood of successfully introducing new modes of action into the market. Therefore, to improve the probability of success, it is essential to explore and increase our understanding of agrodrugs that require improved agrokinetic properties to enhance their effectiveness.

Numerous studies reported in the literature aim to improve the pharmacokinetic properties of agrodrugs by the incorporation of bioactive compounds into delivery systems such as polymeric micelles or nanocarriers [8–10]. The advent of these nanotechnologies enhances bioavailability and promotes targeted delivery; however, many questions remain unresolved on the toxicology and safety of these systems to human and ecosystem health. Furthermore, the high production and formulation costs of agrochemical nanotechnologies currently represent a significant barrier to their widespread industrial adoption [11, 12].

**Fig. 1:**
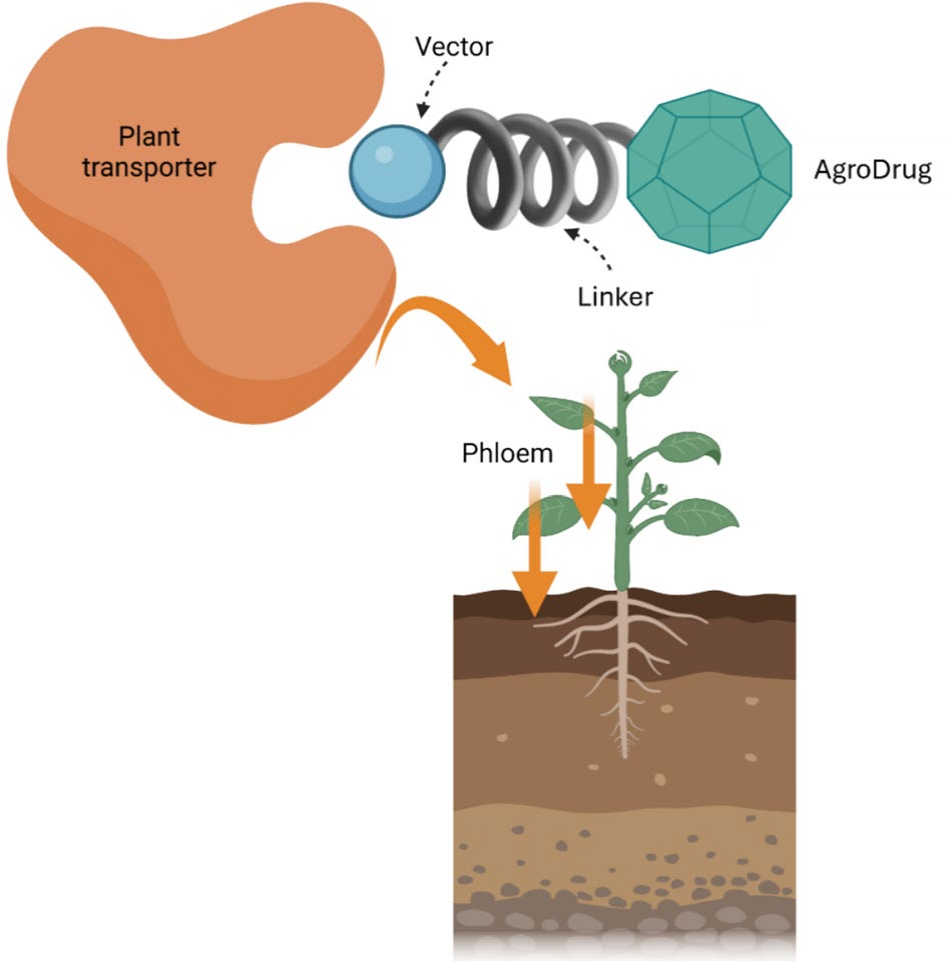
AgroDrug conjugates (AgDCs) are designed to be selectively recognized by a specific transporter and distributed by the phloem system all over the plant. The general structure of AgDCs is represented with the agrodrug as a green octahedron, the vector as a light-blue sphere, and the linker as a gray spring

Another approach widely employed in the pharmaceutical sector is related to the use of drug conjugates, where the bioactive compound is covalently bound to specific carriers or targeting units to enhance either the biodistribution or the selectivity toward a specific tissue of the treated organism. Particularly in the pharmaceutical field, the use of drug conjugate technologies (such as antibody–drug conjugates (ADCs) and small molecule–drug conjugates (SMDCs)) has produced significant therapeutic benefits, especially in oncology [13–16]. To date, there are 15 Food and Drug Administration (FDA)-approved ADCs, and more than 400 ADC candidates are under global development, with over 200 currently in various stages of clinical trials [17]. In addition, around nine SMDCs have advanced to clinical trials, highlighting the rapid evolution and clinical translation of these platforms [18]. A similar application of analogous strategies in crop science relies on the design of AgroDrug conjugates (AgDCs): molecular constructs typically composed of an agrodrug covalently bound to a molecular carrier (named as “vector,” which is generally a sugar derivative or an amino acid) through a linker. This structure potentially enables recognition and active transport by specific cell membrane transporters through the vascular system of plants (Fig. 1).

The attractiveness of AgDCs relies on their claimed ability to enhance biodistribution and improve target engagement [19, 20], which could help to directly reduce the total amount of chemicals needed in the field, minimize off-target dispersion, and limit contamination of soil and water, thus supporting key environmental and EU sustainability objectives [21]. However, despite the conceptual promise of this approach, only a limited and fragmented number of studies have investigated AgDCs so far. Furthermore, from observation of the patent literature and of the reporting of International Organization for Standardization (ISO) common names, there is no clear development candidate coming from these research streams.

This review aims to provide a comprehensive analysis of the various types of AgDCs currently documented in the literature, emphasizing the critical chemical characteristics necessary for their development to advance agrodrug biodistribution and achieve improved crop protection outcomes. Indeed, to date, no systematic effort has been made to analyze these systems from the perspective of their chemical structure, despite growing evidence that structural features play a critical role in plant transporter recognition and compound biodistribution.

By summarizing current knowledge, design principles, and critical challenges, we hope to encourage the scientific community to explore the potential of AgDCs and drive pioneering research that could lead to innovative and effective strategies for achieving more effective and sustainable agriculture.

## Transport Process in Plants

### The Plant Vascular System: Xylem and Phloem

**Fig. 2:**
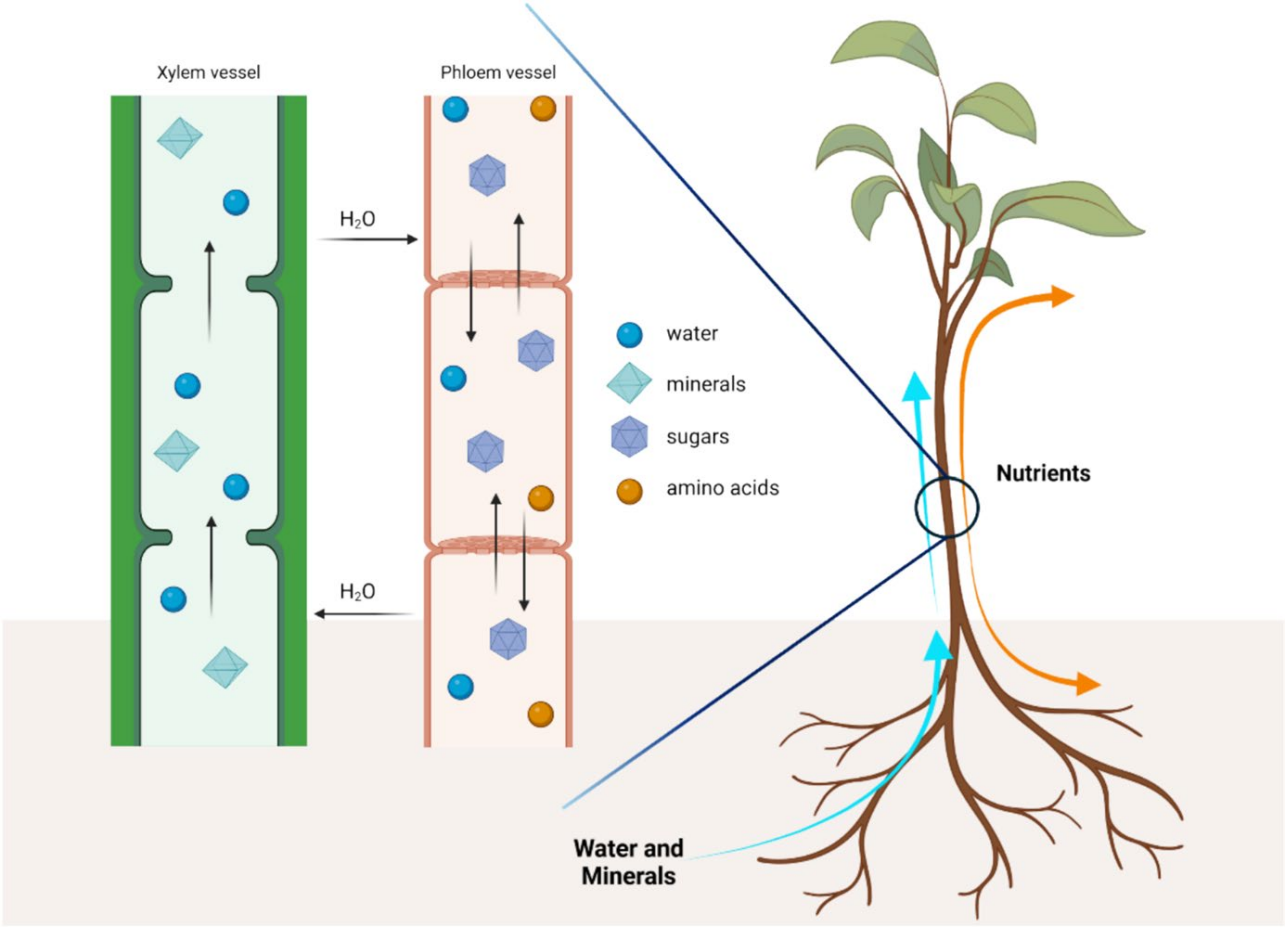
Biodistribution of water, minerals, and nutrients throughout the plant by the xylem and phloem vessels. Nutrients are actively transported from the source cells into the sieve tube elements of the phloem. Because of the high sugar concentration in the phloem, water diffuses from the xylem into the sieve tube elements. When nutrients are transported out of the phloem, water diffuses into the xylem, raising its pressure. These movements of water between the xylem and the phloem systems are, together with the transpiration process, the main mechanisms that control the water pressure and the flow into the xylem vessels

The plant vascular system has been an essential adaptation for plants to thrive on land. It consists of two main components: xylem and phloem. The xylem is responsible for transporting water and dissolved minerals from the roots to the leaves, while the phloem carries energy-rich products, such as sugars and amino acids, from the leaves to nonphotosynthetic parts of the plant such as roots, flowers, and developing structures (Fig. 2).

The main driving force of the transportation process in the xylem is transpiration, which begins with the evaporation of water from the plant’s leaves that creates a negative pressure that finally pulls water up from the roots through the xylem system [22]. Various cell types form the xylem, and, among them, tracheids are the fundamental (and most ancient) cell constituted by a thin elongated hollow dead cell with a lignified cell wall, to minimize water loss during transport, and a central lumen, through which water flows [23]. In addition to tracheids, vessels and fibers are key components in the xylem system. Vessels are composed of multiple vessel elements, which are short, wide, and cylindrical cells arranged end-to-end to form continuous tubes. Owing to their large diameters, vessels are highly efficient in conducting water, also considering the presence of perforations, which allow direct water flow between elements without significant resistance. Fibers, which are prominent in woody plants, are elongated, narrow cells with a small central lumen and lignified secondary thick walls, thereby providing mainly mechanical support to the plant [22]. The translocation process of nutrients by phloem is instead currently explained by Muench’s pressure flow hypothesis [24]. This theory describes how sugars, primarily in the form of sucrose, are transported from sources (where they are produced or stored, such as leaves or storage organs) to sinks (areas of active growth, storage, or metabolism, such as roots, flowers, or fruits). This source-loading and sink-unloading process generates an osmotic pressure gradient, which is the driving force for the distribution of sugars and other molecules throughout plants [19, 25]. For instance, once the nutrient (i.e., the sugar) is loaded into the phloem at the source, the osmotic pressure within the sieve elements increases, causing water to move into the phloem from adjacent xylem vessels by osmosis. This generates a pressure gradient that drives the nutrient along the sieve element toward the sink. At the sink, sugars are removed from the phloem, and the water taken into the phloem at the source returns to the xylem at the sink, completing the cycle and maintaining the flow.

The phloem mainly consists of (i) sieve elements (SEs), elongated cells aligned end to end to form continuous tubes connected by sieve plates with pores that facilitate sap movement; (ii) companion cells (CCs), metabolically active cells adjacent to the SEs that aid in nutrient loading and unloading, and forming a metabolic and genetic unit known as the “sieve element-companion cell complex” (SECCC) through extensive plasmodesmata connections; and (iii) phloem parenchyma cells, which store nutrients and facilitate lateral transport within the phloem tissue [19, 25–27].

### The Plant Transporters

**Fig. 3:**
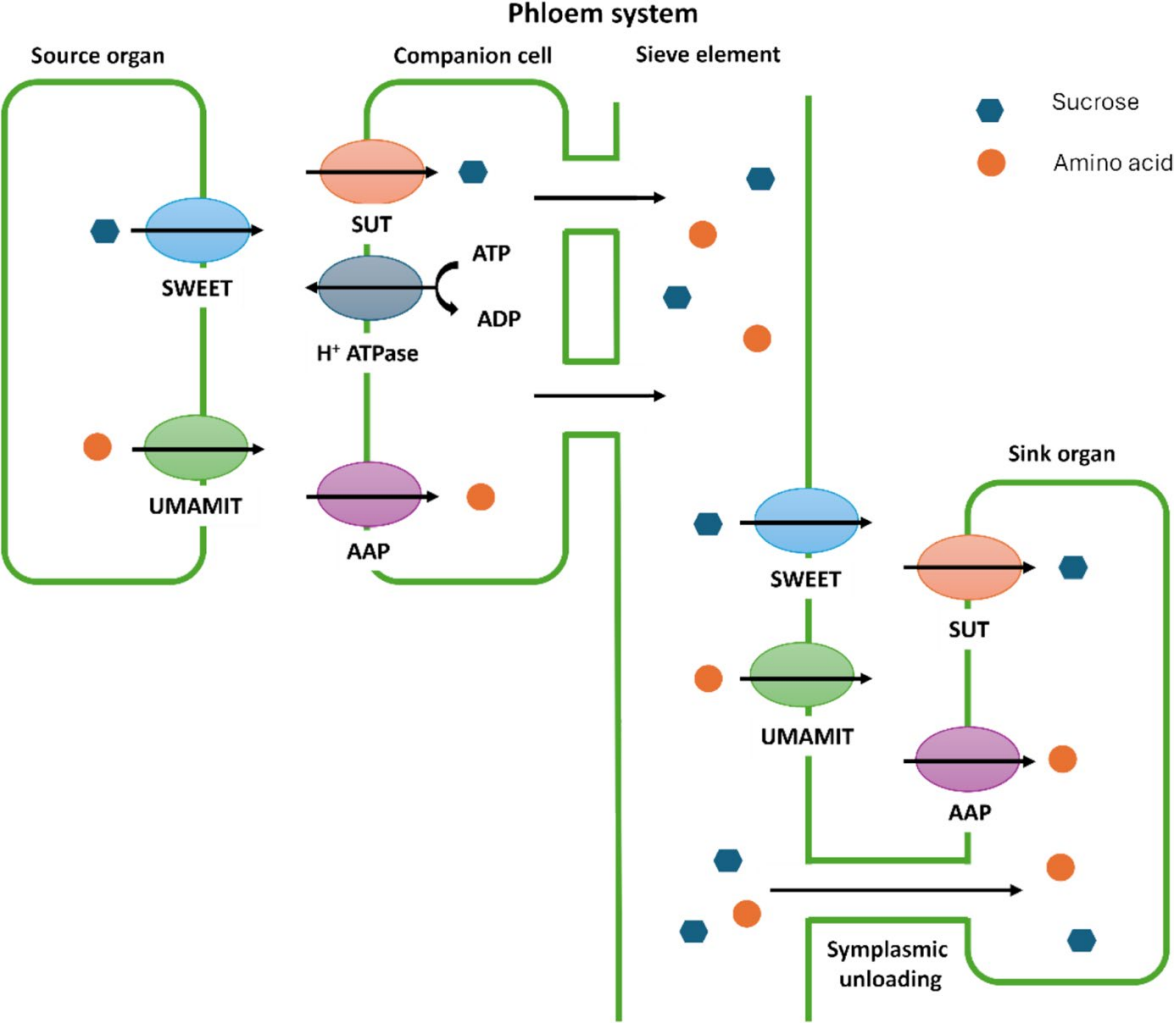
In active apoplasmic loading, nutrients can be released into the apoplasm by efflux carriers (e.g., SWEET for sucrose, UMAMIT for amino acids) before being taken up into the SECCC by plasma membrane-localized transporters (e.g., SUT for sucrose, AAP for amino acids). In the apoplasmic unloading mechanism, the nutrients are unloaded from the SECCC and then loaded into the sink organ by specific transporters; whereas, in symplasmic phloem, unloading the nutrients can diffuse or convect into the sink organ through the abundant plasmodesmata by bulk flow

The bidirectional transport capability of the phloem system makes it an attractive route for delivering agrodrugs to specific tissues or organs within the plant [28–30]. In this context, leveraging nutrients that are naturally transported through the vascular system as carriers for nonsystemic agrodrugs offers a promising strategy to enhance their systemic distribution. Achieving this, however, requires a deep understanding of how nutrients access the vascular system, particularly the phloem. For instance, nutrients must cross the plasma membrane at some point, which is a lipid bilayer structure that surrounds the cell and, in principle, is impermeable to solutes, such as ions and polar molecules. The uptake may involve symplasmic and apoplasmic transport pathways, mediated respectively by passive diffusion or active transporters. The specific transport proteins are embedded in cellular membranes, mediating the entry and exit of molecules or ions by recognizing and binding them in a reversible way [31–33]. Among the various types of transporters present in plants, sugar transporters and amino acids transporters are those that have been exploited to deliver agrodrugs through the phloem systems within an AgDC structure (Fig. 3).

**Fig. 4:**
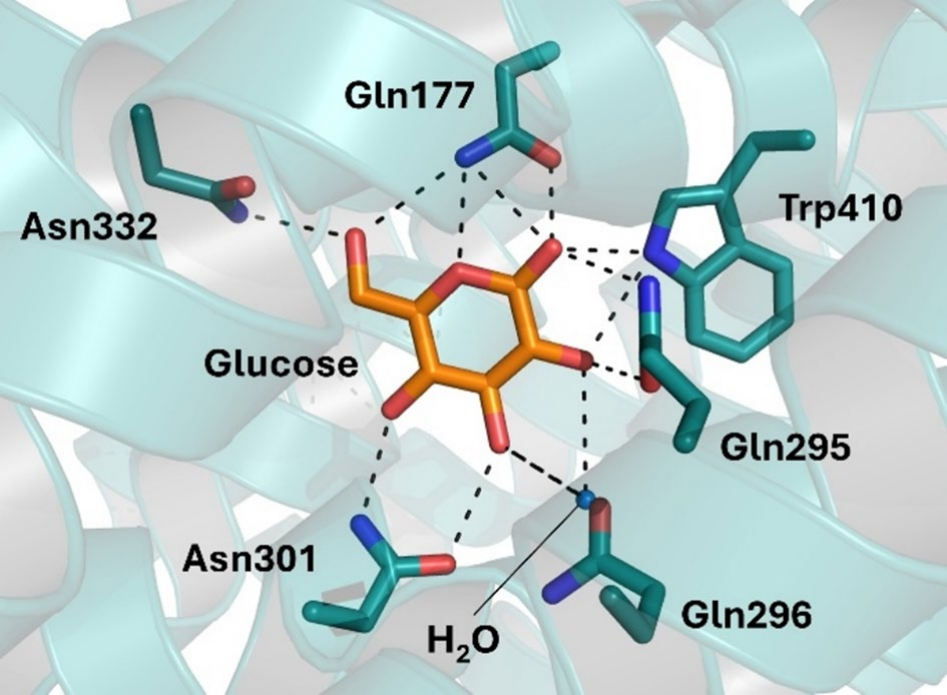
Cocrystal structure of STP10–glucose. The residues of the protein involved in the interaction with glucose are represented in green, whereas the glucose is in orange. The polar interactions are highlighted as dotted lines (PDB: 6H7D)

The most studied sugar transporter families are hexose transporters, such as glucose transporter (GLUT), which facilitate the transport of monosaccharides such as glucose and fructose. These transporters often operate as proton-coupled cotransporters, leveraging the electrochemical proton gradient (H^+^) across membranes to drive sugar uptake against their concentration gradient [34]. Paulsen et al. [35] reported the cocrystal structure of sugar transport protein 10 (STP10)–glucose (Protein Data Bank [PDB]: 6H7D), clarifying fundamental principles of sugar transport in the large monosaccharide transporter (MST) superfamily. In the cocrystal structure, glucose is positioned in the central binding site with well-defined interactions. The C domain of the protein creates a T-shaped CH–π interaction from the residue Phe401 to the main ring of glucose, as well as several polar interactions. The Asn332 residue is in contact with the hydroxyl group of glucose carbon 6 (C6), while a hydrogen bond network is generated between the residues Gln177, Gln295, Gln296, Asn301, and Trp410 and with the C1–C4 hydroxyl groups of glucose (Fig. 4). Meanwhile, the N domain forms a hydrophobic interaction surface for the substrate, facilitating its binding. The polar interactions in the C domain mediate the specificity of STP10, specifically recognizing the hydroxyl groups of the substrate, while the interactions in the N domain primarily regulate the affinity [35].

**Fig. 5:**
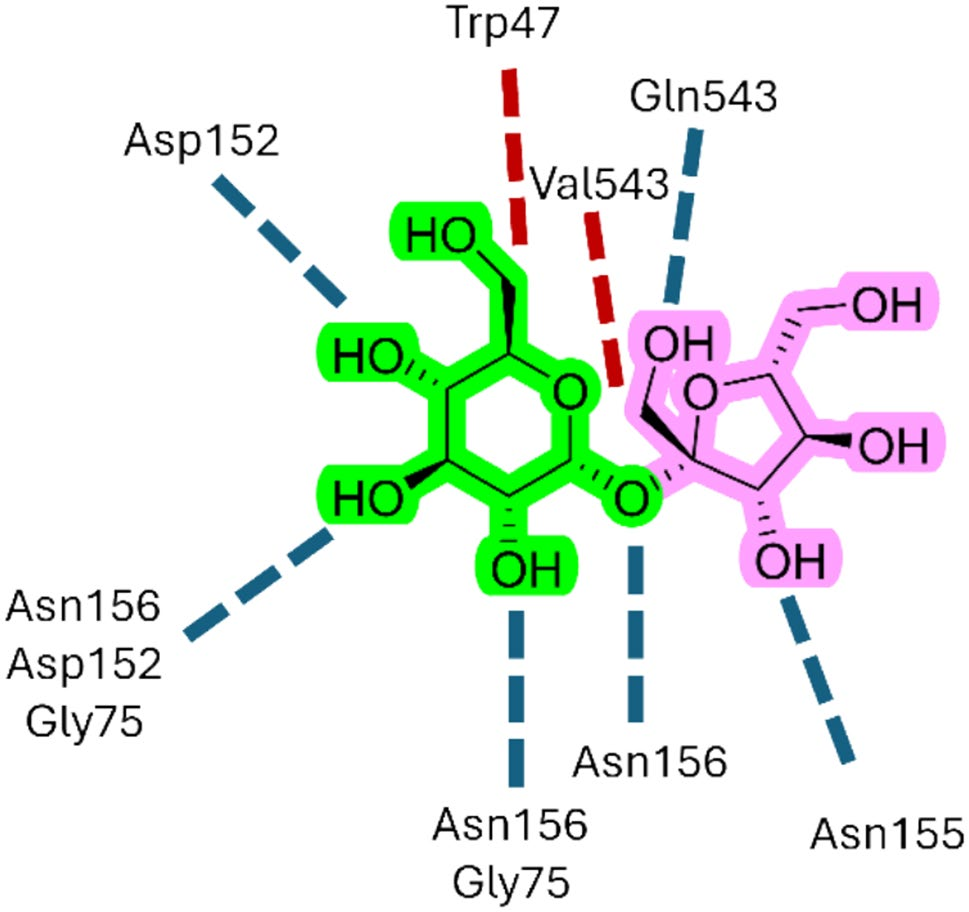
Schematic representation of the sucrose stable pose in simulations with SUC1. Blue dotted lines indicate hydrogen bonds, and red dotted lines indicate hydrophobic interactions. The glucosyl part of the sucrose is highlighted in green, whereas the fructosyl one is in pink

Sucrose is the primary form of sugar transported over long distances: Its loading into the phloem and unloading at sink tissues are critical steps and are primarily mediated by sucrose transporters (SUTs) and sugar will eventually be exported transporters (SWEETs) [36]. Bavnhøj et al. [37] reported the crystal structure of the sucrose–proton symporter *Arabidopsis thaliana* SUC1 (PDB: 8BB6), and performed molecular dynamics (MD) simulations with a sucrose molecule to provide a model for how sucrose is recognized by SUC1 by identifying key structural elements for the substrate binding. Sucrose interacts specifically and directly with the protein by its glucosyl moiety, forming hydrogen bonds between the glucosyl hydroxyls at positions O2, O3, and O4 and the residues Asp152, Gly75, Asn155, and Asn156 (Fig. 5). In addition, hydrophobic interactions contribute to the binding with the glucosyl and, to some extent, also with the fructosyl. Altogether, the simulations identified the glucosyl group of sucrose as the trigger to start the binding event, creating initial selectivity, whereas the recognition of the fructose unit was found to be less important for the transport activity [37].

Beyond sugars, the phloem is also a channel for the remobilization of carbon and nitrogen from source tissues to sink tissues, often in the form of amino acids. These processes occur, for example, during leaf senescence and as a result of de novo amino acid synthesis. Interestingly, all amino acids are found in the phloem, but their levels and composition change constantly, indicating a multitude of mechanisms and transporters involved in their transportation. Amino acid transporters in plants are classified into two primary families on the basis of sequence similarity and uptake characteristics: the amino acid/auxin permease (AAAP) family, also known as the amino acid transporter (ATF) family, and the amino acid-polyamine-organocation (APC) family [38]. The AAAP subfamily is further divided into distinct groups, including general amino acid permeases (GAPs), lysine and histidine transporters (LHTs), γ-aminobutyric acid transporters (GATs), proline transporters (ProTs), indole-3-acetic acid transporters (AUXs), aromatic and neutral amino acid transporters, and amino acid transporter-like proteins. In contrast, the APC family is composed of three subfamilies: cationic amino acid transporters (CATs), amino acid/choline transporters, and polyamine H^+^  symporters (PHSs) [19, 39–42]. In addition, a new group of transporters termed “usually multiple acids move in and out transporters” (UMAMIT) has recently been identified in *Arabidopsis thaliana* [43]. Although there is an existence of many amino acid transporters, to the best of our knowledge, no cocrystal structures are currently available in the literature for the identification of the essential interactions needed for binding. However, several studies have been pursued to understand the type of amino acid transported and the important structural features needed for the recognition [44–46]. For instance, the selectivity for the l-amino acid over the d-amino acid was demonstrated already in the early 1990s [44, 45], and later, Boorer’s group [46] demonstrated the importance of the α-amino portion, in which any modification (i.e., β-alanine and 2-methylamino isobutyric acid were transported poorly, and γ-aminobutyric acid was not recognized at all) leads to a loss of interaction, whereas the substitution of the amino acids in the distal part with both hydrophobic or hydrophilic residues did not influence the recognition.

## General Chemical Structure of AgroDrug Conjugates

**Fig. 6:**
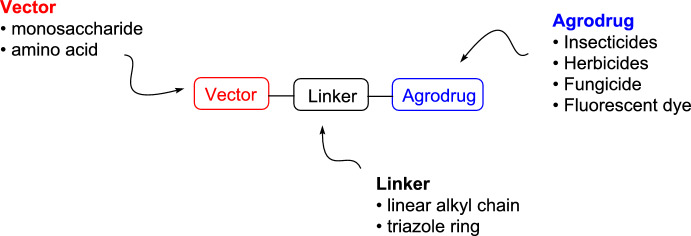
General structure of AgDCs reported in literature

The general structure of these AgDCs is shown in Fig. 6, and it is composed of three units: an agrodrug as payload, which is covalently connected by a linker to a molecular vector capable of being recognized by a specific plant transporter within the plant vascular system.

### Sugar-Based AgDCs

#### d-Glucose–Fipronil Conjugates

In the last decades, several attempts have been made to enhance the phloem mobility of fipronil, a nonsystemic, phenylpyrazole insecticide with broad-spectrum activity against numerous insect pests [47]. One of the major strategies investigated is the conjugation of this agrodrug with d-glucose, which can be transported into the phloem and taken up by hexose transporters.

**Fig. 7:**
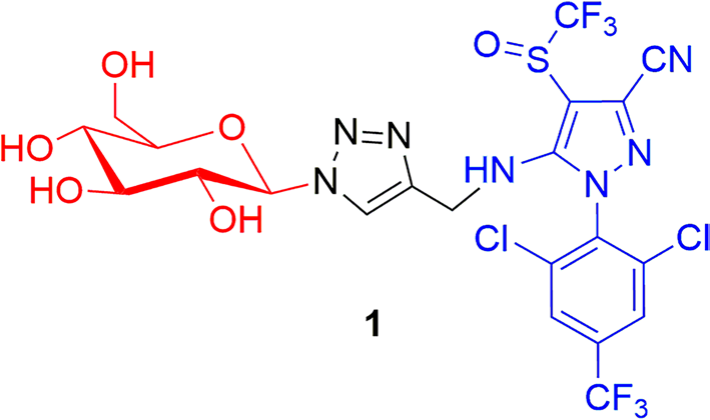
Chemical structures of the β-d-glucose (red)–fipronil (blue) conjugate **1**

In this context, the first example in literature was reported by Yang et al. [48], who synthesized the β-d-glucose conjugate **1** (Fig. 7, Scheme 1).

Compound **1** was identified in the phloem sap after 3 h of incubation with *Ricinus communis* cotyledons. This latter represents the model system extensively used in literature to assess the phloem mobility of nutrients and xenobiotics, also due to the limited availability of standardized assays in other plant species. Owing to the unfavorable physicochemical properties of **1** for passive diffusion across plant membranes (molecular weight [MW] = 680 Da; polar surface area [PSA] = 211 Å^2^, hydrogen bond acceptors [HBA] = 13), the authors hypothesized that an active transport process was involved in its uptake and diffusion through the membranes. Concentration-dependent experiments indicated that the uptake of compound **1** involved two components: a saturable component (i.e. a transporter) at lower concentrations (0.025–0.2 mM), and a nonsaturable component (i.e. passive diffusion mechanism) at higher concentrations [49–51].

**Fig. 8:**
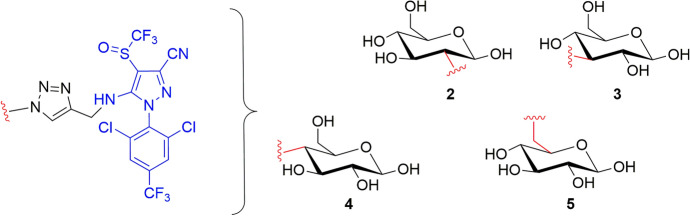
Chemical structures of the β-d-glucose–fipronil conjugates **2–5** in which fipronil is connected to C-2, C-3, C-4, and C-6 (highlighted in red) of β-d-glucose, respectively

The involvement of the monosaccharide transporters was demonstrated by competition experiments in *Ricinus communis*: β-d-Glucose resulted as a stronger competitive ligand of **1** than sucrose, which conversely did not significantly affect the concentration of **1** in the phloem sap [50]. To deeply evaluate the structural features of the glucose moiety to be recognized by the transporters, the effects of the binding between the agrodrug and different positions of glucose were studied by synthesizing four additional derivatives (compounds **2–5**). Interestingly, the analysis of the phloem sap collected after 5 h of incubation with the conjugates showed comparable concentrations among the compounds with a slight difference only for the concentration of compound **4** (concentration in the phloem sap of 32.35 ± 1.51 μM and 26.87 ± 1.07 μM for compound **1** and **4**, respectively), indicating that linking fipronil to C-2, C-3, C-4, and C-6 of β-d-glucose only moderately affected the phloem mobility of the conjugates (Fig. 8, Table 1) [52].

**Table 1:** *R. communis* phloem sap analysis after soaking cotyledons in a solution containing 100 μM of conjugates

Conjugates	Agrodrug	Vector	Concentration (μM)	Reference
1	Fipronil	β-d-Glucose	32.3 ± 1.51^a^	[52]
2	Fipronil	C-2 β-d-glucose	37.4 ± 1.44^a^	[52]
3	Fipronil	C-3 β-d-glucose	35.2 ± 1.32^a^	[52]
4	Fipronil	C-4 β-d-glucose	26.9 ± 1.07^a^	[52]
5	Fipronil	C-6 β-d-glucose	35.1 ± 1.19^a^	[52]
7	Fipronil	β-d-Glucose	23.0 ± 1.9^b^	[56]
11	Fipronil	β-d-Fucose	27.3 ± 2.1^b^	[57]
12	Fipronil	α-l-Rhamnose	27.3 ± 1.9^b^	[57]
13	Fipronil	α-d-Arabinose	21.8 ± 1.9^b^	[57]
14	Fipronil	β-d-Xylose	15.3 ± 2.2^b^	[57]
15	Fipronil	β-l-Fucose	10.6 ± 1.8^b^	[57]
16	Fipronil	α-d-Mannose	7.6 ± 1.6^b^	[57]
17	Fipronil	α-d-Rhamnose	6.1 ± 1.4^b^	[57]
18	Fipronil	β-d-Galactose	ND^b^	[57]
19	Fipronil	β-d-Deoxyglucose	ND^b^	[57]
20	Rotenone	β-d-Glucose	4.9 ± 0.6^c^	[59]
21	Rotenone	β-d-Galactose	ND^c^	[59]
22	Rotenone	α-d-Mannose	ND^c^	[59]
23	Rotenone	β-d-Xylose	ND^c^	[59]
24	Rotenone	α-d-Arabinose	ND^c^	[59]
25	Tralopyril	β-d-Glucose	4.0^d^	[60]
26	Tralopyril	Methyl glucuronate	1.5^d^	[60]
27	Tralopyril	Glucuronic acid	40.0^d^	[60]
28	Tralopyril	β-d-glucose	2.5^d^	[60]
29	Tralopyril	Methyl glucuronate	1.0^d^	[60]
30	Tralopyril	Glucuronic acid	50.0^d^	[60]

^a^The data were acquired after 5 h of incubation [52];^b^after 2 h of incubation [57];^c^after 6 h of incubation [59];^d^after 3 h of incubation with 200 μM of conjugate [60]. ND= not detected

**Fig. 9:**
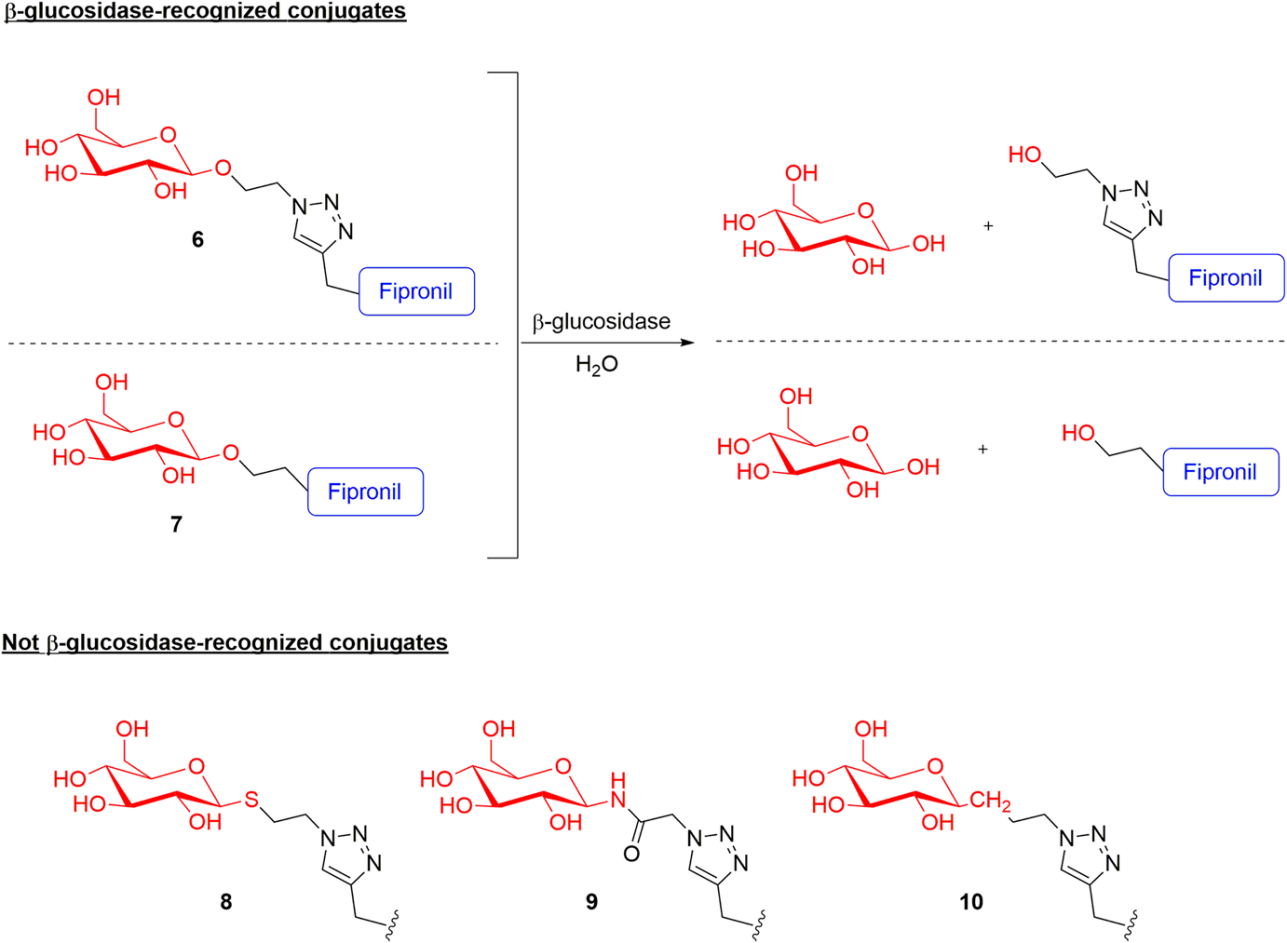
Chemical structures of the d-glucose–fipronil conjugates **6–10** in which β-d-glucose is connected to the rest of conjugate through *O*-, *N*-, *S*-, and *C*-glycosidic bonds, respectively

Since the expression of endogenous β-glucosidase in plants is well known [53–55], Xia et al. [56] investigated the susceptibility of the glucose-based conjugates to release the fipronil upon β-glucosidase enzyme activity. For instance, five conjugates were synthesized (compounds **6–10**, Fig. 9), in which the fipronil-triazole derivative was connected to *O*-, *N*-, *S*-, and *C*-glycosidic bonds. Both the in vitro assay with almond β-glucosidase and the in vivo test conducted in adult *Ricinus communis* indicated high specificity for the *O*-glycosidic bond. Indeed, the hydrolysis of the glycosidic bond was observed only for compounds **6** and **7**. In contrast, compounds **8**, **9**, and **10** (characterized by *S*-, *N*-, and *C*- glycosidic bonds, respectively) were shown to be stable up to 4 h after injection [56].

#### Additional Monosaccharide–Fipronil Conjugates

Given that the monosaccharide transporter superfamily consists of many members, and several of these proteins can transport more than one monosaccharide, further studies were performed evaluating the phloem mobility of fipronil conjugates with different monosaccharides (hexoses, pentoses, and 6-deoxysugars), linked with the *O*-glycosidic bond [57].

**Fig. 10:**
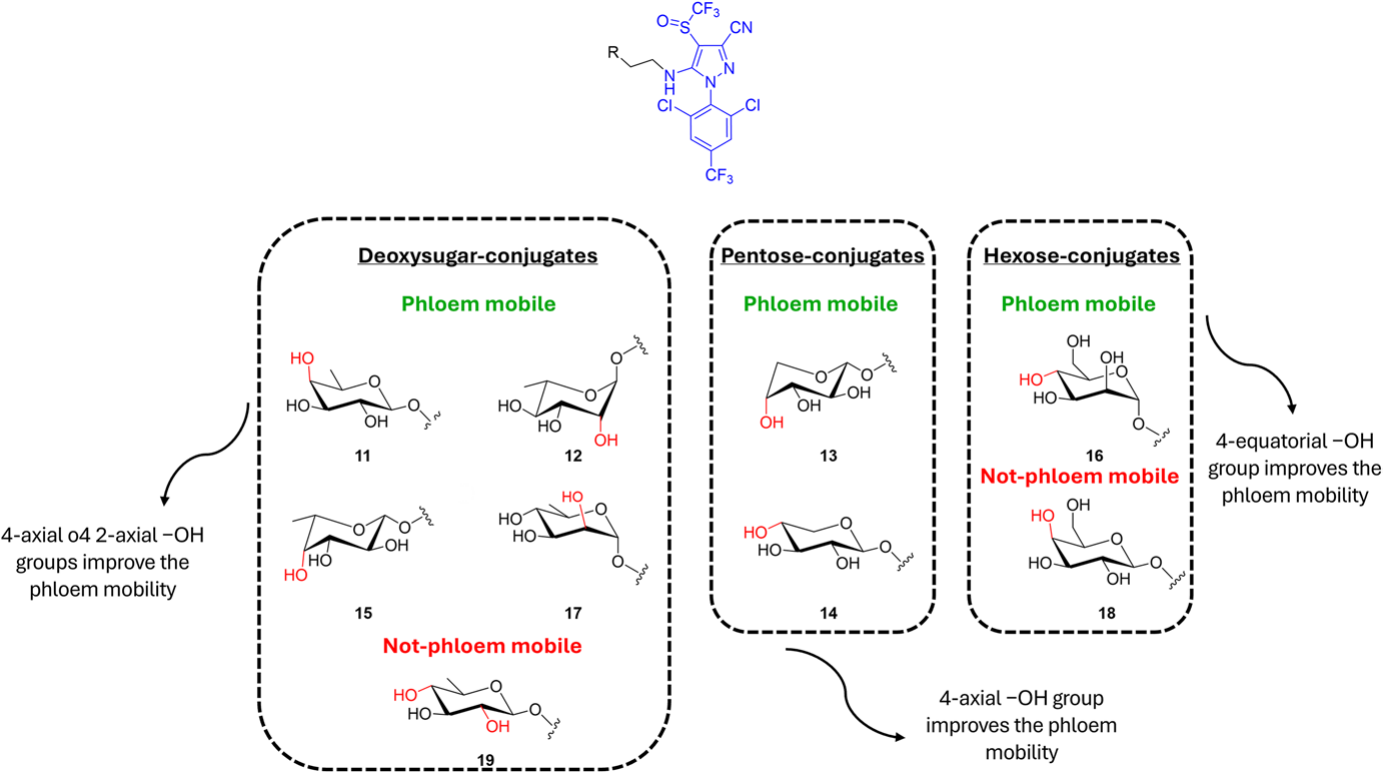
Monosaccharide–fipronil (blue) conjugates **11–19** divided into the different classes of monosaccharides and in phloem and not-phloem mobile conjugates. The differences among the positions of the −OH groups are highlighted in red

Among the tested conjugates, the 6-deoxysugar-based conjugates β-d-fucose–fipronil (compound **11**) and α-l-rhamnose–fipronil (compound **12**) exhibited the best phloem mobility (concentration in the phloem sap after the incubation of 100 μM of the conjugates was 27.3 μM for both **11** and **12**) (Fig. 10, Table 1). By contrast, the β-d-deoxyglucose–fipronil (**19**) and the hexose β-d-galactose–fipronil (**18**) conjugates were not detected at all, thereby suggesting that these conjugates are not recognized and transported into the phloem by specific transporters. The observed differences in the phloem mobility among the three classes of monosaccharides can be used to make a few general considerations of the necessary chemical features to improve the conjugate biodistribution for each category. For instance, the fact that the deoxysugar−fipronil conjugate **19** exhibited no phloem mobility, whereas derivatives **11** and **12** demonstrated to be the most active, may highlight the importance of the 4-axial −OH or 2-axial −OH groups of the 6-deoxysugar for the phloem mobility. However, for the pentose conjugates, α-d-arabinose–fipronil (**13**) exhibited better phloem mobility than β-d-xylose–fipronil (**14**), suggesting that the 4-axial −OH group of pentose may improve phloem mobility. Finally, among the hexose conjugates, the nonmobility of **18** indicates that the 4-equatorial −OH group of hexoses may help with transporter recognition and hence improve the biodistribution via the phloem (Fig. 10) [57].

#### Payload Miscellaneous

**Fig. 11:**
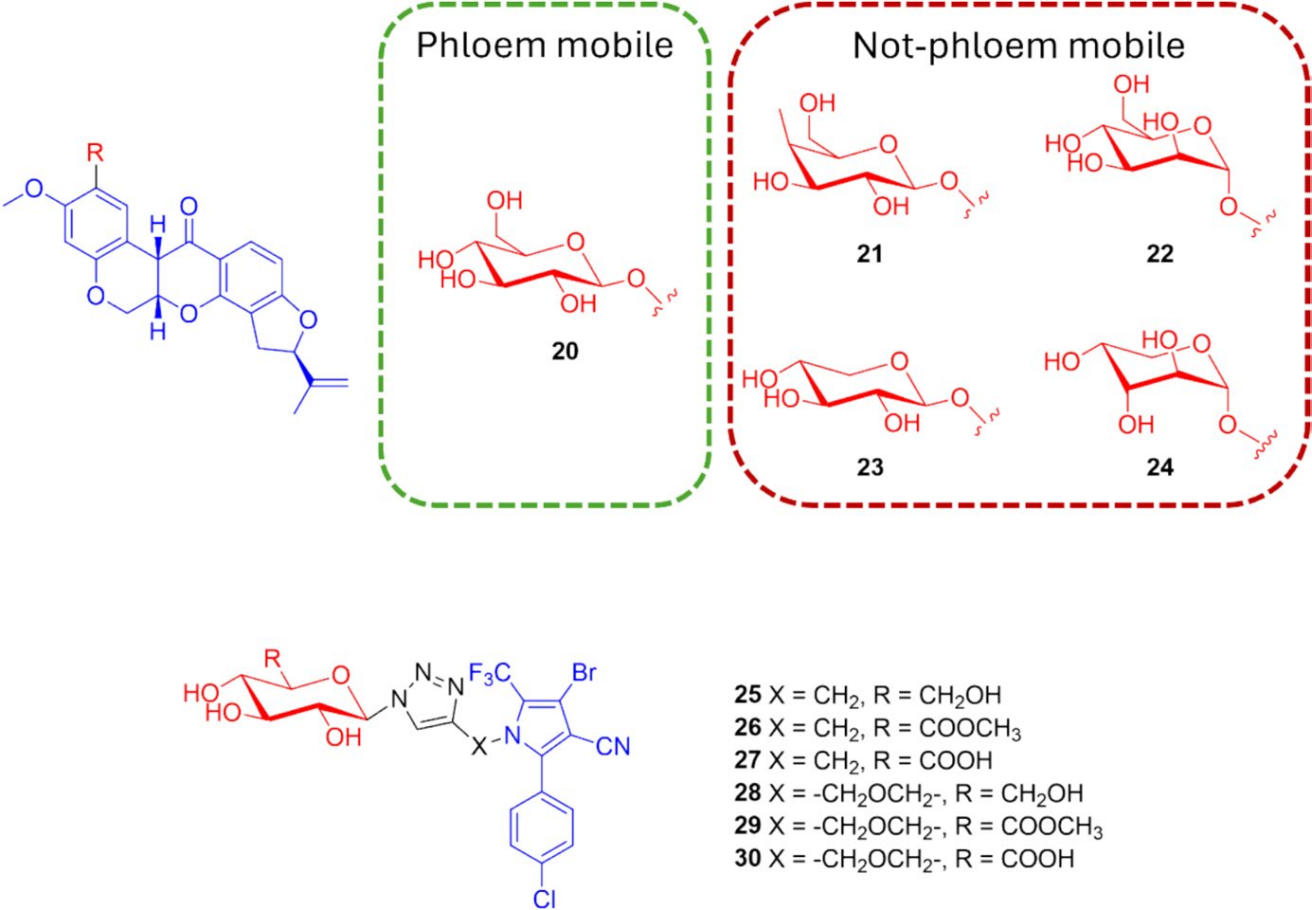
Chemical structures of the monosaccharide-based (red) conjugates of rotenone (blue) **20–24**, and of tralopyril (blue) **25–30**. Monosaccaride = β-d-glucose (**20**, **25**, **28**), β-d-galactose (**21**), α-d-mannose (**22**), β-d-xylose (**23**), α-d-arabinose (**24**), methyl glucuronate (**26**, **29**), and glucuronic acid (**27**, **30**)

To understand whether the mobility of the monosaccharide conjugates could also be affected by the selected agrodrug, several sugars were linked by an *O*-glycosidic bond to rotenone, a natural insecticide obtained from the root extracts of some leguminous plants such as *Derris* and *Lonchocarpus* [58]. Differing from the data previously observed for the fipronil conjugates described above [57], the agrokinetic experiment results showed that only β-d-glucose–rotenone (**20**) exhibited phloem mobility, whereas conjugates bearing the vectors β-d-galactose (**21**), α-d-mannose (**22**), β-d-xylose (**23**), and α-d-arabinose (**24**), respectively, were not found (Fig. 11). Thus, the agrodrug biodistribution can be effectively improved through a coupling with a monosaccharide, but the extent of phloem mobility is also affected by the parent molecule [59].

This evidence was proved also by the study of Chen et al. [60], who connected the agrodrug tralopyril and its prodrug to β-d-glucose through the 1,2,3-triazole moieties (compounds **25–27** and compound **28–30**, respectively, Fig. 11). The concentrations in the *Ricinus communis* phloem sap of the so-obtained conjugates **25** and **28** were approximately 0.1-fold greater than that of the fipronil analogue **1** after 3 h of incubation. The authors also tried to modify the monosaccharide moiety by introducing methyl glucuronate (compounds **26** and **29**) or glucuronic acid moieties (compounds **27** and **30**). The concentration in the phloem sap of conjugates **27** and **30**, characterized by the glucuronic acid, was approximately 10-fold and 20-fold higher than that of conjugates **25** and **28** bearing the β-d-glucose, and 27-fold and 50-fold greater than that of conjugates **26** and **29**, characterized by methyl glucuronate. These results indicate that the carboxyl group of the glucuronic acid has a positive contribution to phloem mobility, probably due to the translocation of conjugates by “phloem ion-tapping” (Fig. 11) [60].

The phloem sap analysis data of the sugar-based conjugates are presented in Table 1.

**Fig. 12:**
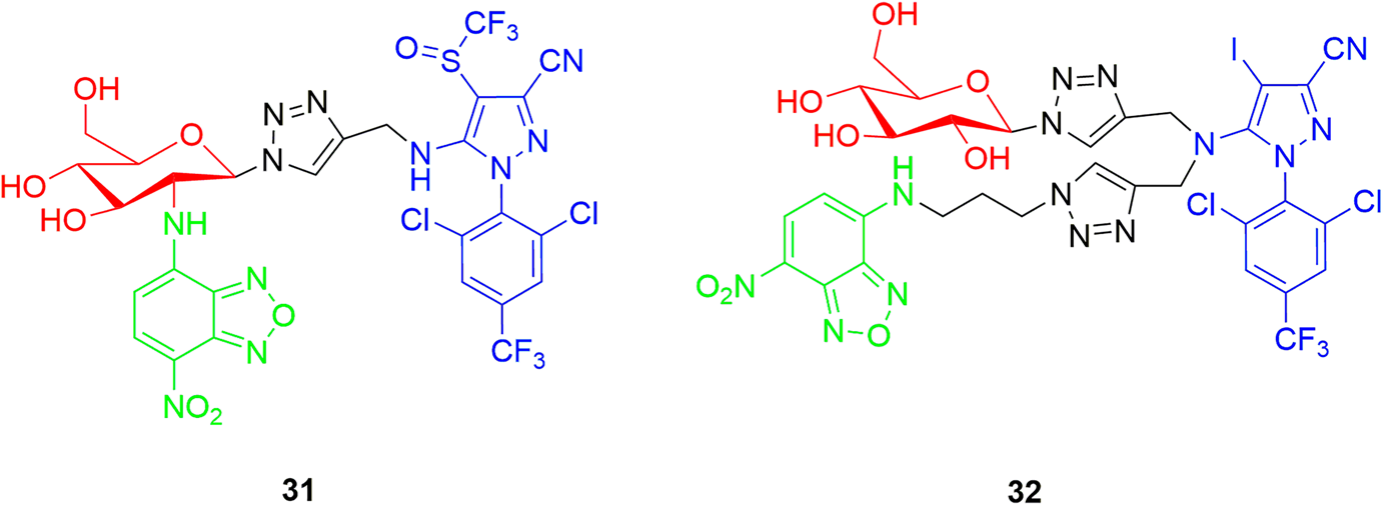
Chemical structures of the fluorescent conjugates **31** and **32**. Red: d-glucose; blue: the agrodrug; green: the fluorescent dye

To further investigate the uptake pathways, transport routes, and distributions of xenobiotics within plants, studies based on fluorescence tagging techniques were explored to provide dynamic and real-time information on xenobiotics at the cellular level [61–63]. More specifically, the β-d-glucose-based conjugate of fipronil (**31**), or its analog 4-iodo-1-phenylpyrazole (**32**), were labeled with the 7-nitrobenz-2-oxa-1,3-diazole (NBD) fluorescent tag (Fig. 12) [64–66].

The uptake and transport of **31** and **32** within castor bean seedlings was performed using confocal laser scanning microscopy. The results of imaging experiments showed that these fluorescent conjugates could be loaded into sieve tubes after transiting through epidermal cells and mesophyll cells, and therefore, they could be translocated over the entire plant via the phloem system [64–66]. However, these results could also be affected by the different physicochemical properties of fluorescent derivatives compared with the original conjugates. Indeed, these differences could play a significant role in determining how a compound moves through plant tissues, interacts with cellular structures, and accumulates in specific regions.

### Amino Acid-Based AgDCs

**Fig. 13:**
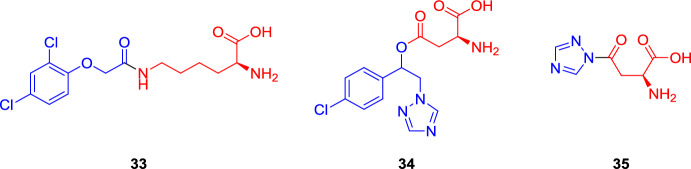
Chemical structures of the amino acid-based conjugates **33–35**. The vectors are highlighted in red, whereas the agrodrugs are highlighted in blue

The ability of amino acid transporters to recognize agrodrugs functionalized with an α-amino acid group was first demonstrated by Dufaud et al. in the 1990s [67]. In particular, they conjugated the herbicide 2,4-dichlorophenoxyacetic acid to the amino group of the lysine side chain (**33**), and the fungicides 1-(4-chlorophenyl)-2-[(1*H*)-1,2,4-triazol-1-yl]ethanol and 1,2,4-triazole to the carboxyl functionality of the aspartic acid side chain (conjugate **34** and **35**, respectively) (Fig. 13).

The following uptake studies performed on broad bean plants incubated with a 5 mM solution of conjugates **33** and **35,** and with a 2 mM solution of conjugate **34**, demonstrated how the uptake of the isotopic labeled [^3^H]threonine was inhibited up to 90% by compound **33** displaying the lysine as the vector, and up to 55% by conjugate **34** bearing the aspartic acid. By contrast, derivative **35** did not affect the uptake of natural amino acid, highlighting that, although the α-amino acid group proved to be essential for recognition between the substrate and the transporter, it did not appear to be sufficient. Interestingly, the d-lysine derivative of compound **33** had practically no effect on the uptake of neutral, acidic, and basic amino acids. Only tryptophan and phenylalanine inhibited **33** uptake, hence suggesting the involvement of their transporters in the recognition and transportation of the conjugate [49, 67]. Finally, compound **33** demonstrated to be phloem mobile in broad bean (*Vicia faba*), and it exhibited a different distribution pattern in the whole plant compared with the free agrodrug, especially featuring a greater efficiency in reaching the apical part of the roots. In addition, a low amount of the free drug was identified in the phloem sap after the incubation of **33** (concentration ≈1/8 of **33** absorbed by leaf tissues after 5 h), suggesting that the conjugate can progressively be hydrolyzed within the tissues to lysine and the free agrodrug [49].

**Fig. 14:**
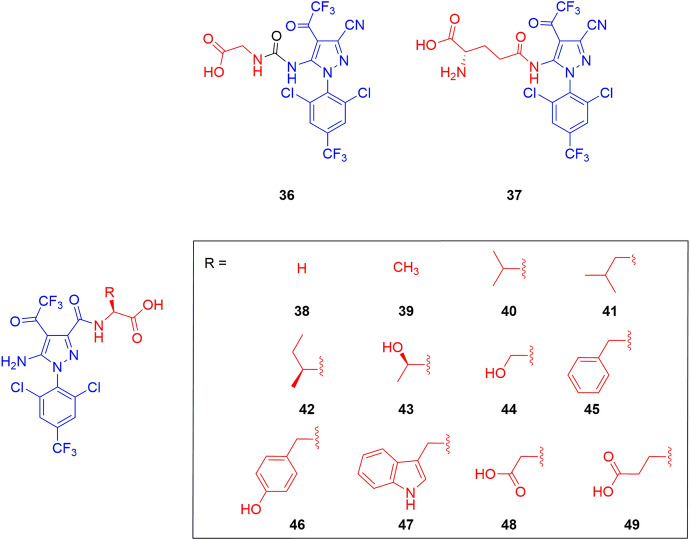
Chemical structures of the fipronil–amino acid conjugates **36–49**. The vectors are highlighted in red and fipronil in blue

From these first examples, various AgDCs displaying different payloads were screened for their phloem mobility once connected to a specific amino acid. For example, Sheng et al. conjugated the insecticide fipronil to glycine (**36**, Fig. 14) and to glutamine (**37**, Fig. 14) at the amine residue of the pyrazole ring, and then they explored a different conjugation site with a wider series of amino acids (compounds **38–49**, Fig. 14) [68, 69].

In general, competition experiments demonstrated the involvement of active transporters also in these cases. In addition, compound **37** displaying a glutamine-based vector resulted in a significantly improved phloem mobility in *R. communis* seedlings compared with the glycine-based conjugate **36** [68]. In a later work, the authors explored a different conjugation site of fipronil. The conjugation at the carboxylic group on the pyrazole ring present in compounds **38–49** (Fig. 14) seemed to be preferred for better phloem mobility, underlining the importance of considering not only the type of amino acids used as vector but also the specific points in the agrodrug’s chemical structure where these amino acids are attached [69]. In particular, conjugate **38** showed a higher phloem concentration compared with conjugate **36** having the aromatic amine of fipronil as the conjugation site, although both are conjugated to a glycine residue (concentration after 6 h of incubation of 15.00 ± 2.50 and 10.14 ± 0.30 for **38** and **36**, respectively). Conjugate **44**, characterized by the serine residue, showed the best phloem mobility results among this series of derivatives [69].

**Fig. 15:**
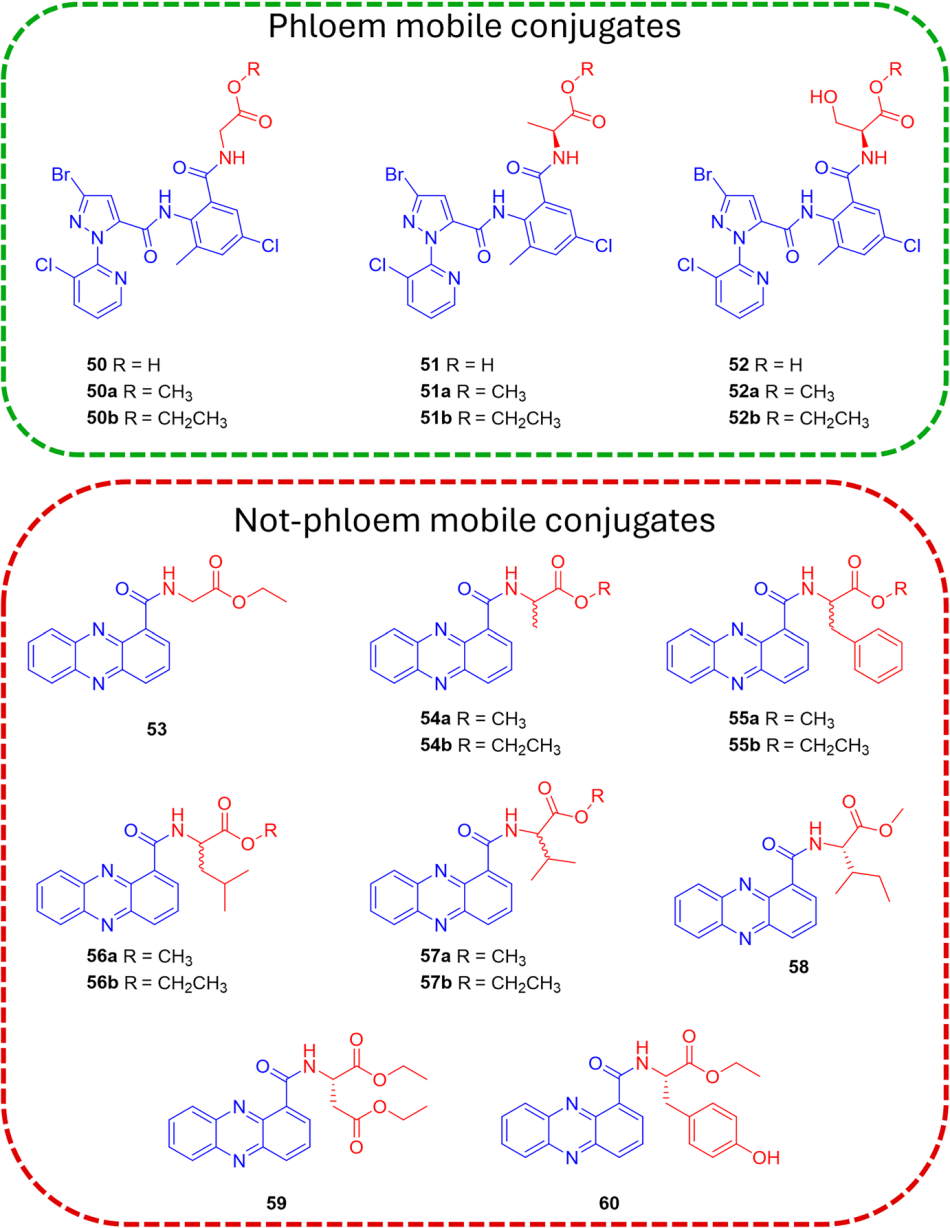
Chemical structures of chlorantraniliprole (blue) conjugate with glycine (**50**), alanine (**51**), and serine (**52**) residues (red) and their corresponding methyl and ethyl ester derivatives, and of phenazine-1-carboxylic acid (PCA) conjugate with the ester derivatives of several amino acids (**53–60**). Compounds without indicated stereochemistry were prepared with both l- and d-amino acid forms

However, this positive result about the serine-conjugate **44** was in contrast with the findings of Yao et al., who conjugated the non-phloem mobile insecticide of the ryanoid class chlorantraniliprole to several amino acids (compounds **50–52**, Fig. 15) to investigate the possibility of improving its biodistribution [70]. Indeed, the phloem mobility of compounds **50** and **51** (characterized by the conjugation of chlorantraniliprole with glycine and alanine residues, respectively) in *Ricinus communis* showed to be better than serine conjugate **52** [70], highlighting that phloem mobility could be highly affected by the physicochemical property of the agrodrug also in the case of amino acid-based conjugates.

Yao and colleagues also explored the impact of protecting the carboxylic function of the amino acid vector as methyl and ethyl esters (compound **50a,b**–**52a,b**). With their increased lipophilicity, the ester derivatives may enhance the passive diffusion of the conjugates across the membrane. Then upon uptake in the plant, endogenous esterases may hydrolyze the esters, enabling successful recognition by amino acid transporters. As hypothesized, in cotyledon incubation experiments, the ester derivatives showed significantly higher amounts of uptake compared with the amino acid conjugates in the carboxylic form, and led to the accumulation of the hydrolyzed forms **50** and **51** in phloem tissues. Compound **51b** exhibited the highest concentration under the applied experimental conditions (313.57 ± 16.08 μm at 5 h) in the form of its hydrolyzed product **51**, which was 3.1-fold that in the incubation medium [70].

The positive influence of the ester derivatization obtained with the chlorantraniliprole conjugates needs to be considered again with a substrate-dependent attitude, as demonstrated by Niu et al. in their attempt to improve the biodistribution of phenazine-1-carboxylic acid (PCA) through the conjugation of ester derivatives of a series of amino acids (compounds **53–60**, Fig. 15) [71].

**Fig. 16:**
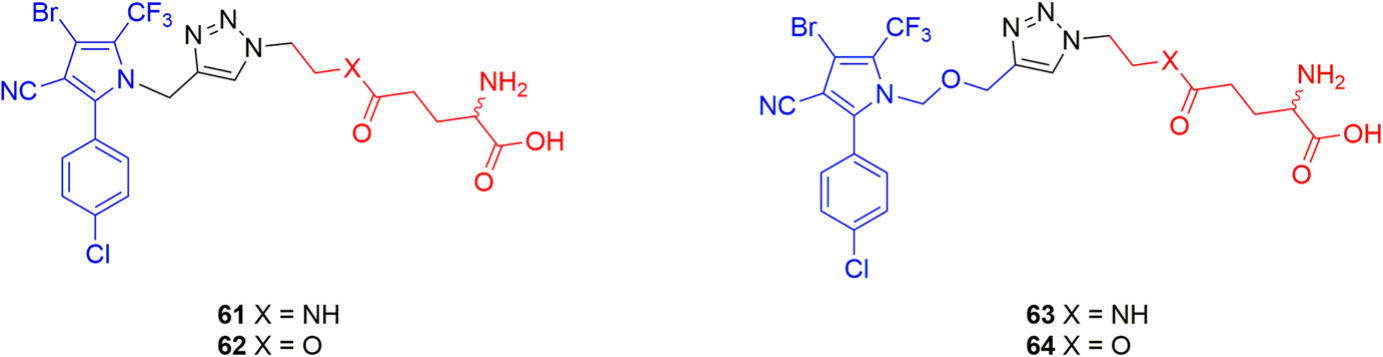
Chemical structures of α-amino acid (red) conjugates **61–64**. Tralopyril and its prodrug are highlighted in blue. The stereochemistry of the amino acids was not specified in the original paper

Besides the traditional amino acids, Yang et al. compared the effect on biodistribution of noncommon amino acids as vectors [72]. In particular, the non-phloem mobile insecticide tralopyril and its prodrug version called chlorfenapyr were functionalized with theanine (compounds **61** and **63**), a unique nonproteic amino acid found almost exclusively in tea, and with glutamic acid (compounds **62** and **64**) (Fig. 16). The incubation and phloem sap collection experiments showed the presence of conjugates **61**–**64** with a slightly better efficiency for the ones characterized by the theanine residue [72].

The results of *R. communis* phloem sap analysis of conjugates **36–64** are summarized in Table 2.

**Table 2:** *R. communis* phloem sap analysis after soaking cotyledons in a solution containing the specified conjugate

Conjugates	Agrodrug	Vector	Concentration (μM)	References
36	Fipronil	Glycine	10.1 ± 0.3^a^	[69]
L-37	Fipronil	l-Glutamine	38.0 ± 2.2^b^	[69]
D-37	Fipronil	d-Glutamine	20.0 ± 1.2^c^	[69]
38	Fipronil	Glycine	15.0 ± 2.5^a^	[69]
39	Fipronil	Alanine	35.0 ± 4.0^a^	[69]
40	Fipronil	Valine	27.0 ± 4.0^a^	[69]
41	Fipronil	Leucine	15.0 ± 1.5^a^	[69]
42	Fipronil	Isoleucine	ND^a^	[69]
43	Fipronil	Threonine	18.0 ± 2.6^a^	[69]
44	Fipronil	Serine	52.0 ± 5.8^a^	[69]
45	Fipronil	Phenylalanine	6.1 ± 0.8^a^	[69]
46	Fipronil	Tyrosine	12.0 ± 2.0^a^	[69]
47	Fipronil	Tryptophan	8.7 ± 2.0^a^	[69]
48	Fipronil	Aspartic acid	41.8 ± 5.0^a^	[69]
49	Fipronil	Glutamic acid	20.0 ± 3.0^a^	[69]
50a	Chlorantraniliprole	Glycine methyl ester	160^d^	[70]
51a	Chlorantraniliprole	Alanine methyl ester	260^d^	[70]
51b	Chlorantraniliprole	Alanine ethyl ester	313.6 ± 16.1^d^	[70]
61	Tralopyril	Theanine	150^e^	[72]
62	Tralopyril	Glutamic acid	120^e^	[72]
63	Chlorfenapyr	Theanine	60^e^	[72]
64	Chlorfenapy	Glutamic acid	55^e^	[72]

Phloem sap collection started 5 h after the beginning of soaking. Samples were incubated with: ^a^100 μM of conjugate, collection after 6 h of incubation [69]; ^b^50 μM of conjugate [69]; ^c^200 μM of conjugate [69]; ^d^100 μM of conjugate; the compounds were detected in the acidic form [70]; ^e^200 μM of conjugate, after 3 h of incubation [72]. If not specified, the amino acid residues are in the natural L isoform. ND = not detected

### Direct Comparison of Sugar-Based and Amino Acid-Based Conjugates

**Fig. 17:**
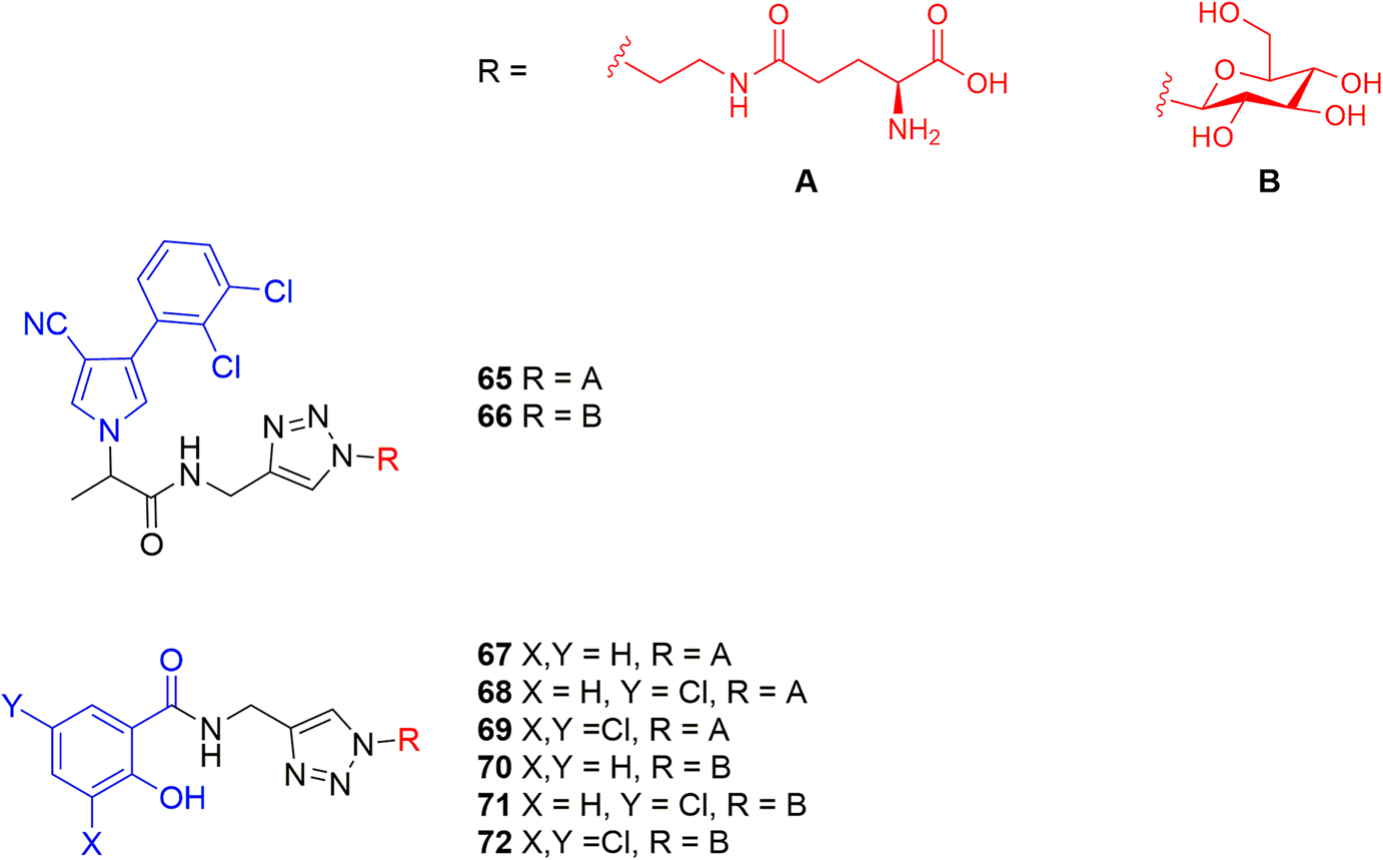
Chemical structures of agrodrug (blue) conjugates with l-glutamic acid (compounds **65**, **67–69**) or β-D-glucose (compounds **66**, **70–71**). The vectors are highlighted in red

Wu et al. [73] directly compared the ability of amino acid and sugar carrier systems to translocate large agrodrugs. An acidic derivative of the fungicide fenpiclonil, previously developed by Chollet et al. [74], was selected as the model system and functionalized with l-glutamic acid or β-D-glucose (compounds **65** and **66**, respectively, Fig. 17). An hour after the beginning of incubation of *Ricinus communis* cotyledons in the standard solution with compound **65** at a concentration of 100 μM, the amino acid conjugate was found in the phloem sap. Its concentration increased up to 5 h to reach about 12 μM. By contrast, the phloem systemicity of compound **66** was extremely low. Its concentration in the phloem sap was about 20 times lower than that of compound **65** [73].

Similarly, the study of Guichard et al. [75] indicated that the amino acid-based conjugates of salicylic acid derivatives (compounds **67–69**, Fig. 17) showed better phloem mobility properties than the sugar-based conjugates (compounds **70–72**, Fig. 17). However, given that salicylic acid and its monochlorinated analogs in position 5 or dichlorinated ones in positions 3 and 5 already showed good phloem mobility properties, the conjugates did not improve their biodistribution. Indeed, **67** and **69** showed almost the same level of phloem mobility as their parent compounds, whereas the phloem mobility of glucose conjugates (**70–72**) displayed much lower levels [75].

This comparative approach between sugars and amino acids offers valuable insights for designing AgroDrug conjugates tailored to target specific plant transport mechanisms. Overall, in these studies, amino acids were found to be more promising carriers than sugars in enhancing the phloem loading of AgroDrug conjugates [73, 75].

### Linker

The design of the linker is a critical factor for enhancing the delivery, stability, and specificity of AgDCs within plant systems. The linker is needed to covalently connect the vector with the agrodrug, which could already bear a suitable conjugation site or, if that is not the case, a preliminary modification is necessary by altering a substituent or introducing an appropriate functional group.

Generally, for the AgDCs, the linkers can be mainly divided into two chemical groups: the triazole-based ones and the linear aliphatic chain.

**Fig. 18:**
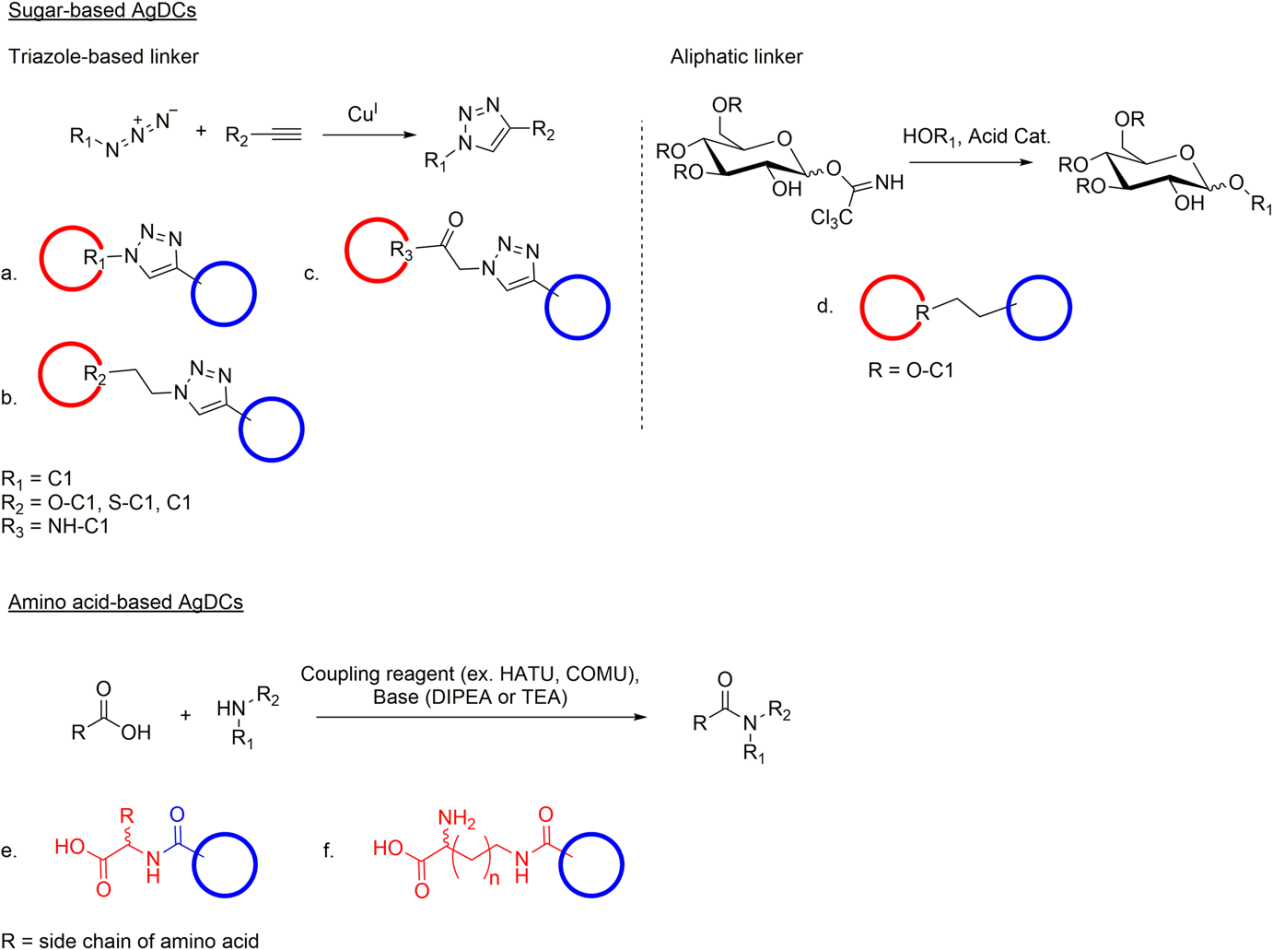
General scheme of: (**a–c**) the copper-catalyzed azide-alkyne cycloaddition reaction used for the synthesis of sugar-based AgDCs with a triazole linker; (**d**) trichloroacetimidate glycosylation used for the synthesis of sugar-based AgDCs with an aliphatic linker; (**e–f**) amide coupling used for the synthesis of amino acid-based AgDCs. The agrodrugs are represented with a blue circle and the sugar vectors with a red circle, whereas the general structure of the amino acid vectors is highlighted in red. HATU, 1-[*bis*(dimethylamino)methylene]-1*H*-1,2,3-triazolo[4,5-*b*]pyridinium 3-oxide hexafluorophosphate; COMU, 1-cyano-2-ethoxy-2-oxoethylidenaminooxy; DIPEA, *N,N*-diisopropylethylamine; TEA, triethylamine

In the sugar-based AgDCs, the linkers based on a 1,2,3-triazole scaffold have been used more often than the linear aliphatic chain owing to their ability to undergo these cycloaddition reactions (i.e., copper-catalyzed azide-alkyne cycloaddition) in aqueous or mild conditions, which is beneficial for maintaining the integrity of the rest of the molecule (Fig. 18a–c) [48, 50, 56, 72, 75–77]. In the case of aliphatic linkers (Fig. 18d), the alkyl chain can be linked to the sugar by a glycosidic bond through the trichloroacetimidate method [57].

By contrast, the amino acid vectors are generally connected to the payload through direct coupling between the α-amine of the amino acid residue, and a carboxylic residue already present in the structure of the agrodrug or previously introduced by preliminary functionalization (i.e., PCA and fipronil conjugates, respectively, Fig. 18e) [71]. Alternatively, the carboxylic acid residue of the side chain of the amino acids (glutamic acid and aspartic acid) can be used to perform the amide coupling reaction with an agrodrug featuring an amino functionality, such as in the case of fipronil (Fig. 18f) [68].

**Fig. 19:**
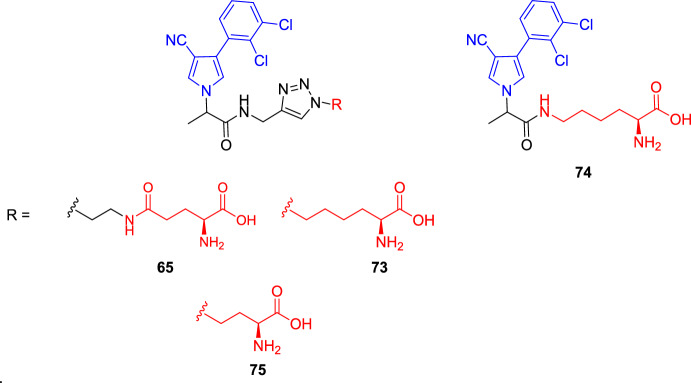
Chemical structure of fenpiclonil conjugate compounds **65** and **73–75**

Marhadour et al. [78] investigated the impact of the two different types of linkers by comparing the phloem concentration of fenpiclonil conjugates with and without the triazole moiety (compounds **73** and **74**, respectively, Fig. 19). Their findings indicated that the triazole spacer did not affect the systemicity of the conjugates in *Ricinus communis* cotyledons. In addition, a comparison between conjugates **65**, **73**, and **75** (Fig. 19), which contain the same triazole spacer but differ in the length of the carbon chain of the l-amino acid, indicated that the reduction of the latter led to increased phloem mobility, probably due to less steric hindrance (concentration after 6 h of incubation with 100 μM of conjugate ≈ 15 μM for **65**; 20 μM for **73** and **74**; and 30 μM for **75**) [78].

.

### Critical Insights into the Development and Biological Evaluation of AgDCs

To fully understand the potential and limitations of AgDCs, it is essential to discuss in more detail their final effect on the selected plant models, as well as to consider additional factors that may affect their practical applicability.

For example, one important factor to take into account is that, in most cases, the phloem mobile assessment of the synthesized AgDCs was evaluated in vitro or in ex vivo assays, which is often a good indicator but is not always fully transferable to higher tiered studies.

**Fig. 20:**
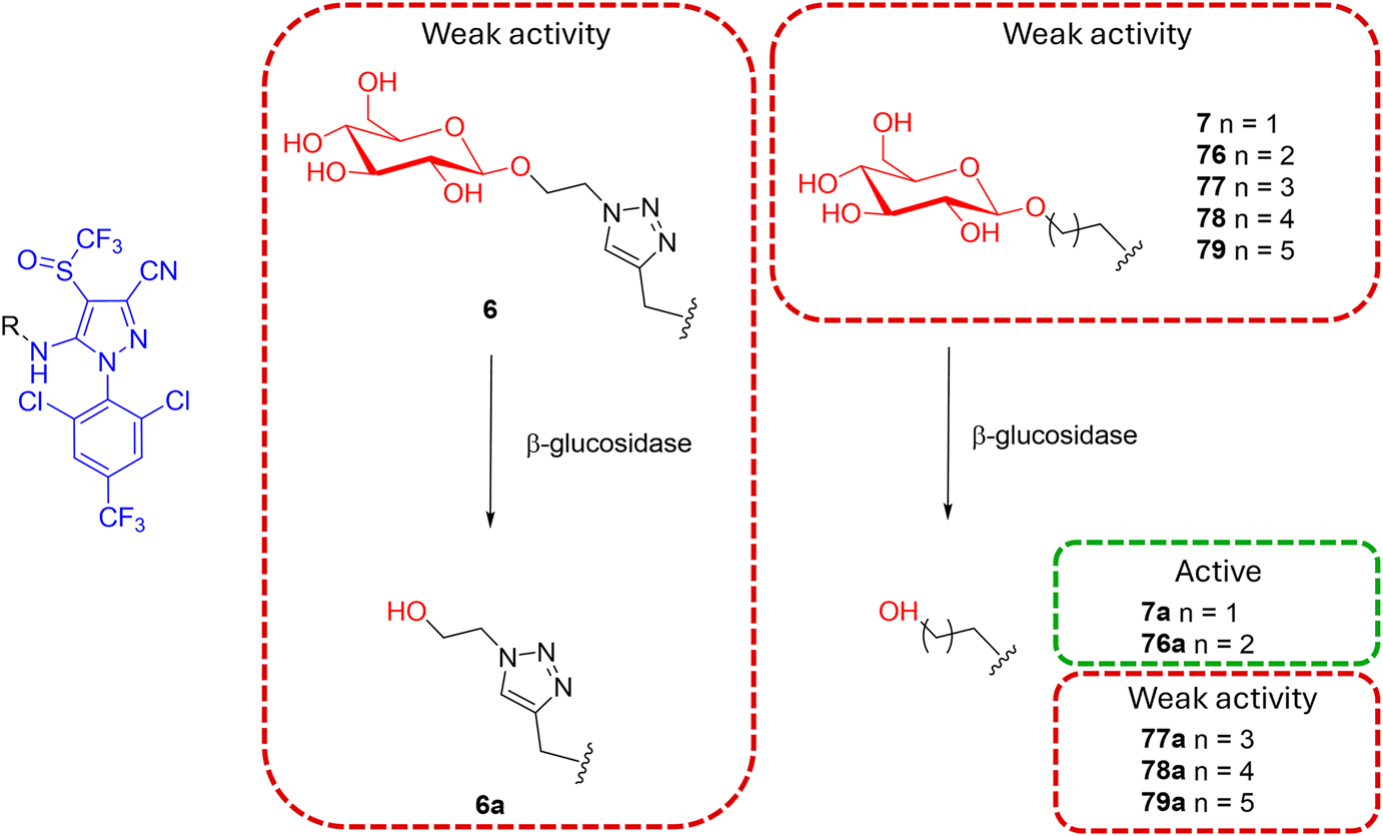
Chemical structures of AgroDrug conjugates **6**, **7**, and **76–79**, and of their metabolites **6a**, **7a**, and **76a–79a**

This limitation may also have influenced the final biological activity that was observed. For instance, even if the in vitro or ex vivo identification of phloem-mobile AgDCs was successful, their subsequent biological evaluation revealed that almost all tested conjugates showed markedly reduced potency compared with their parent compounds. [48, 50, 56, 57, 70, 73, 75, 79]. One of the few derivatives that showed comparable activity to that of the free fipronil using leaf-dipping technique was compound **7** (Fig. 20), which displayed a good, but not the highest (Table 1), phloem concentration after incubation with *R. communis* cotyledons (lethal concentration 50% [LC_50_] values after 24 h against the larvae of *P. xylostella* L.; and 31.49 mg L^−1^ and 25.50 mg L^−1^ for **7a** and fipronil, respectively; LC_50_ values against the second-instar larvae of *S. litura* F. of 36.34 mg L^−1^ and 33.87 mg L^−1^ for **7a** and fipronil, respectively) [56]. Such a good outcome was referred to the production of the less bulky metabolite **7a** compared with the whole conjugate upon β-glucosidase enzyme proteolytic activity. Probably, the introduction of bulky substituents on fipronil can induce a drastic reduction of activity due to steric hindrance issues in the binding pocket of the biological target. The unsatisfactory insecticidal activity at 500 mg L^−1^ of the metabolite of compound **6** (**6a**, Fig. 20), characterized by the fipronil bound only to the triazole liker (mortality rates of only 30% against *P. xylostella* L. and 9.7% against *S. litura* F. after 24 h), seems to confirm this hypothesis [56]. However, no phloem concentration data are reported for this AgDC.

A similar trend was observed by Wen and co-authors [80]. Indeed, the insecticidal activity of the metabolite **76a** was comparable to that of the commercial insecticide (mortality rate of *P. xylostella* larvae after incubation for 24 h with the compounds at concentrations of 0.63 mg L^−1^ of 82%, and 76% for fipronil and **76a**, respectively), whereas metabolites **77a**, **78a**, and **79a**, characterized by bulkier linkers, exhibited weak insecticidal activities, with mortality rates against *P. xylostella* after 24 h of 48% for **77a**, and < 30% for **78a**, and **79a** (Fig. 20). Once again, no phloem mobility assay data are available for these latter compounds.

It is therefore evident that the efficacy assessment of AgDCs requires further development and refinement. Moreover, several additional limitations should be acknowledged. First, in-plant metabolic instability may lead to reduced bioavailability or altered degradation pathways, which in turn can significantly affect the expected insecticidal performance. Furthermore, there are potential toxicity concerns regarding linker fragments; this would be mitigated by utilizing linker fragments that are ideally already known to not exhibit any adverse toxicological effects and that are readily degradable in the environment to ensure that they do not persist and accumulate. For novel linker entities lacking toxicity data, it would be prudent to prioritize only those fragments without predicted adverse effects.

It should also be considered that increasing molecular weight and synthetic complexity can elevate the cost of goods (CoGs). While AgDCs will generally be more expensive than their parent active ingredients, comparisons with newly developed active ingredients may, in some cases, still result in favorable CoGs.

The lack of field-scale validation remains a significant hurdle. The transition from greenhouse to field trials introduces numerous environmental variables, often requiring multiple optimization cycles before achieving consistent performance. Current research has also been largely limited to a narrow range of crop species, restricting broader applicability, so expanding studies to diverse plant systems will be crucial to fully understand the versatility and limitations of AgDC technology. Finally, as AgDC research progresses toward potential commercialization, it will also be essential to consider its long-term ecological fate and safety. This includes a thorough investigation of degradation pathways, environmental persistence, and potential toxicity of all the components.

Taken together, these aspects point to the need for a more holistic evaluation of AgDCs that goes beyond systemicity and potency, integrating metabolic fate, environmental safety, economic viability, and field-level efficacy.

## Conclusions and Outlooks

The agrochemical industry is poised for significant advancements in crop protection, driven by the urgent need for innovative solutions to combat increasing pest resistance and environmental challenges. Recent innovations in crop protection research highlight the importance of developing new modes of action that can effectively address these issues while promoting sustainable agricultural practices. The ability to enhance the effectiveness of new potential products through the use of conjugates may play a crucial role in achieving these goals. By improving how these agrodrugs are absorbed and distributed within plants, conjugates can increase their efficacy and reduce the need for higher chemical inputs. Indeed, the effectiveness of an agrodrug is intricately linked to its ability to be absorbed and distributed within plant tissues, similar to how the pharmacokinetic profile determines a drug’s success in human therapy. This review highlights AgroDrug conjugates (AgDCs) as an innovative strategy to enhance agrodrug biodistribution and reduce environmental impact. However, this approach faces several challenges, and, to date, the interplay between chemical modifications, vector conjugation, and the overall effectiveness of agrodrugs remains complex and requires further investigations. Moving forward, we suggest that a balanced approach is needed, one that optimizes vector selection, conjugation sites, and linker chemistry to refine both phloem mobility and bioactivity. Many of the reported AgDCs show improved bioavailability and systemic translocation but often suffer from reduced potency due to structural derivatization. A possibility of limiting the effect of derivatization could be the exploitation of physiological triggers such as endogenous β-glucosidase that could enable the targeted release of active compounds within plants [55, 81, 82]. In addition, optimizing phloem mobility requires a systematic evaluation of the vector selection and the specific structural site for conjugation, as these factors are critical for effective transport and distribution within plant tissues.

AgDCs are still in their infancy, and they have a long way to go until they can make an impact on the crop protection market. Only with more time and research will other advanced factors, such as field trial validation and applicability for broad-spectrum use, become clearer. We expect that there will be significant challenges to overcome in the field, with many optimization cycles being needed, but every new AgDC tested would provide useful insights into their behavior and provide hypotheses on how to overcome the observed issues. A scientifically cautious approach, supported by comprehensive environmental and toxicological studies, will be essential to ensure that AgDCs evolve into safe, sustainable, and effective solutions in modern crop protection strategies.

## Data Availability

No datasets were generated or analyzed during the current study.
